# Who Is Best to Test? A Systematic Review of Chlamydia Infections in Switzerland

**DOI:** 10.3390/ijerph17249389

**Published:** 2020-12-15

**Authors:** Melanie Haag, Elisabeth Zemp, Kurt E. Hersberger, Isabelle Arnet

**Affiliations:** 1 Pharmaceutical Care Research Group, University of Basel, 4001 Basel, Switzerland; kurt.hersberger@unibas.ch (K.E.H.); isabelle.arnet@unibas.ch (I.A.); 2 Swiss Tropical and Public Health Institute, Socinstrasse 57, 4002 Basel, Switzerland; elisabeth.zemp@swisstph.ch; 3 University of Basel, 4001 Basel, Switzerland

**Keywords:** chlamydia, screening, pharmacy, Switzerland

## Abstract

In many countries, community pharmacies provide sexual-health-related services to limit the spread of sexually transmitted infections (STIs), including chlamydia testing. To identify suitable target groups for pharmacy-based chlamydia testing in Switzerland, we aimed to assess chlamydia prevalence, identify risk groups, and delineate screening strategies. We conducted a systematic literature search up to December 2019 in PubMed, EMBASE, and Web of Science, according to the PRISMA guidelines, using as keywords “chlamydia”, “screening”, and “Switzerland”. Two researchers screened the title, abstract, and full-text article and assessed the methodological quality. The literature search generated 108 hits, and nine studies were included. Chlamydia prevalence ranged between 0.8 and 12.8%. Most frequently affected were undocumented women undergoing voluntary termination of pregnancy (12.8%, 95% CI: 8.4–18.9), HIV-positive men who have sex with men (10.9%, 95% CI: 9.2–17.6), and adult offenders (6.5%, 95% CI: 3.2–9.0). Systematic screening was suggested for the first two risk groups and women suffering a miscarriage. To conclude, chlamydia infections are prevalent in Switzerland, but the identified risk groups are difficult to reach for a pharmacy-based testing service. More studies are needed to identify suitable target groups, including customers seeking sexual health services, particularly emergency contraception users who already receive counselling for STIs at community pharmacies.

## 1. Introduction

In many countries, community pharmacies offer sexual health-related services including the provision of emergency contraception, regular hormonal contraception and condoms, and dispensing of misoprostol for medical abortion [1]. Some countries have also evaluated pharmacy-based testing for sexually transmitted infections (STIs) such as human immunodeficiency virus (HIV) and chlamydia infections to limit the spread [1,2]. So far, England has been the only country that has implemented pharmacy-based chlamydia testing into a national chlamydia-screening programme [3]. A systematic review and meta-analysis showed a pooled chlamydia positivity rate of patients screened in community pharmacies of 8.1% (95% CI: 7.3–8.9%), mainly consisting of emergency contraception (EC) users [4]. EC users were also at increased risk for chlamydia infections in a Scottish family planning clinic, where EC users under 24 years had a chlamydia prevalence rate of 7.6% [5]. EC users fulfil many risk factors [6], which may include young age (<25 years), being female, having a new sexual partner, having more than one sexual partner, prior STI, and inconsistent use of condoms [7].

Pharmacy-based testing for sexually transmitted infections focused primarily on chlamydia because it is the most frequently sexually transmitted infection (STI) in high-income countries, including Switzerland [8,9]. Nationally, chlamydia infections peak in women aged 15–24 years and in men aged 25–34 years [9]. The infection is asymptomatic in up to 95% of affected women and more than 50% of affected men [10]. If untreated, chlamydia can clear spontaneously, persist, or ascend to the upper genital tract, where it can cause pelvic inflammatory disease (PID) [11]. PID can lead to scarring in fallopian tubes, which is a contributing factor for chronic pelvic pain, tubal infertility, ectopic pregnancy in women, and epididymitis in men [7,10]. The American Centre for Disease Control and Prevention recommends annual chlamydia screening of sexually active women below 25 years of age and older women at increased risk of infection [12]. The Swiss Federal Office of Public Health did not adopt this recommendation. According to the current Swiss guidelines, chlamydia testing is indicated when symptoms occur, another STI is diagnosed, the partner has a confirmed STI, before termination of pregnancy (ToP), and before inserting an intrauterine device [13].

Swiss community pharmacies may increase access to chlamydia testing, as they have long opening hours, widespread locations, and pharmacists who are seen as professional and competent providers [14,15]. Thus, we aimed to identify suitable target groups for pharmacy-based chlamydia testing according to the Swiss prevalence data. We conducted a systematic literature search to determine chlamydia prevalence rates, identify risk groups, and delineate chlamydia-testing strategies.

## 2. Materials and Methods

### 2.1. Search Method

We performed a systematic literature search according to the PRISMA guidelines [16]. Three electronic databases, PubMed, EMBASE, and Web of Science were searched up to 17 December 2019. The search was restricted to humans, with no language restrictions. The search strategy combined the following terms: chlamydia [MeSH] OR Chlamydia trachomatis [MeSH] OR chlamydia infection [MeSH] AND screening OR mass screening [MeSH] OR testing OR diagnosis AND Switzerland [MeSH] OR Swiss (Appendix A).

### 2.2. Study Selection and Data Collection

The search results were merged into Endnote X9 and duplicates removed. Studies reporting on exclusive *Chlamydia trachomatis* screening in Switzerland were included. Exclusion criteria were reporting on STIs in general, on a diagnostic test procedure, on *Chlamydia trachomatis* in animals, the use of official register data, congress abstracts, and letters to the editor. Two independent reviewers (M.H., I.A.) screened the title, abstract, and full-text article. Bilateral discussions resolved discrepancies. Data extraction comprised study design, venue of testing, analytical sample, analytical test, participation rate, chlamydia prevalence, authors’ conclusions, and patients’ characteristics such as gender and mean age. We disregarded a subgroup of female prisoners in one study as only 20 women were recruited compared to 214 men [17].

### 2.3. Quality Assessment

We assessed the methodological quality and the risk of bias of the included studies with the U.S. National Institutes of Health’s quality assessment tool for observational cohort and cross-sectional studies [18]. Researchers (M.H., I.A.) answered each item with “yes”, “no”, or “not applicable (N/A)”. (Appendix B). A score adapted from Sommer et al. [19] was created to categorise the methodological quality into good, fair, and poor. Items answered with “yes” were awarded one point, while items answered with “no” or “not reported” got zero points. The sum was divided by the number of items answered with “yes”, “no”, or “not reported”, disregarding “N/A” answers. Depending on the score, the quality was good (score 0.75–1), fair (score 0.5–0.75), or poor (score < 0.5). A participation rate of eligible individuals was calculated, with the number of individuals enrolled in the study as the numerator, and the number of eligible individuals asked to participate as the denominator. No response rate was calculated for studies where the required numbers were not reported.

### 2.4. Statistical Analysis

Prevalence data and the 95% confidence interval (CI) were used as reported in the included article. We calculated 95% CI using the prop. test in RStudio (version 1.1.463, Boston, MA, USA) when it was missing in the study [20].

## 3. Results

**Figure 1:**
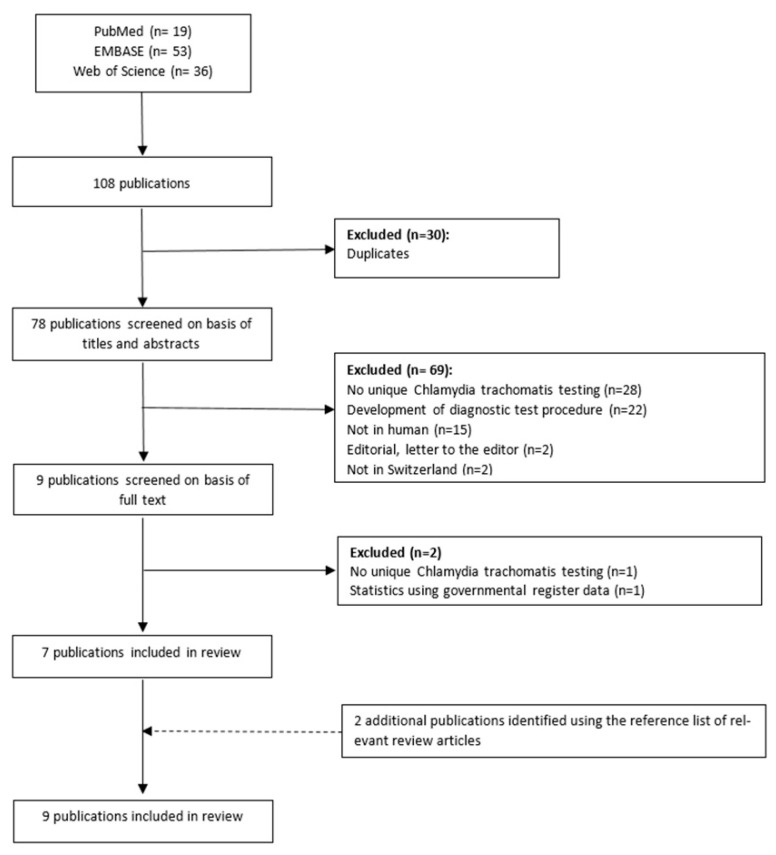
The selection process of the included articles.

Of the 108 retrieved records, 78 remained after removing duplicates. Of these, 69 were excluded because they did not meet the inclusion criteria based on the title and abstract. The full text of nine articles was examined. Two of these were excluded because they did not fulfil the inclusion criteria. Two additional studies were identified by checking the reference lists [21,22]. Finally, nine studies contributed to the review (Figure 1).

In total, 6049 people received chlamydia screening. Studies took place in different language regions including francophone regions only (*n* = 6) [17,21,23,24,25,26], francophone and germanophone regions (*n* = 1) [27], and multiple language regions by means of national networks (*n* = 2) [22,28]. One study examining Swiss men as part of mandatory army recruitment was conducted at the population level, whereas eight studies targeted specific risk groups. Recruitment sites were university hospitals [21,26], outpatient clinics [27,28], private medical practices [22], an army recruitment centre [23], a community mobile care unit [25], cantonal sexual health network centres [27], and detention facilities [17,24]. Table 1 summarises the characteristics of the included studies.

### 3.1. Study Population

Studied populations consisted of HIV positive men who have sex with men (MSM) [28], offenders [17,24], recruits [23], undocumented immigrants [25,26], young patients attending a cantonal sexual health network centre [27], and women seeking gynaecological care [21,22,26]. The latter included women at their first pregnancy consultation [22], regular check-up [22], having a miscarriage [21], undergoing voluntary termination of pregnancy (ToP) [26], or having an uneventful pregnancy [21]. Overall, the participants’ mean age ranged from 16 to 42 years [24,28]. Within comparable risk groups, participants differed in age, with adolescent offenders being 10 years younger than adult offenders (mean: 16.2 years vs. 26.4 years), and in origin, with adolescent offenders being more frequently from European countries compared to adult offenders (60% vs. 31.6%) [17,24]. Moreover, women seeking gynaecological care were on average > 25 years of age. Their origin differed in that 87% of women attending a first pregnancy consultation were Swiss compared to 99.4% of undocumented women undergoing ToP were non-European [22].

### 3.2. Chlamydia Prevalence

**Figure 2:**
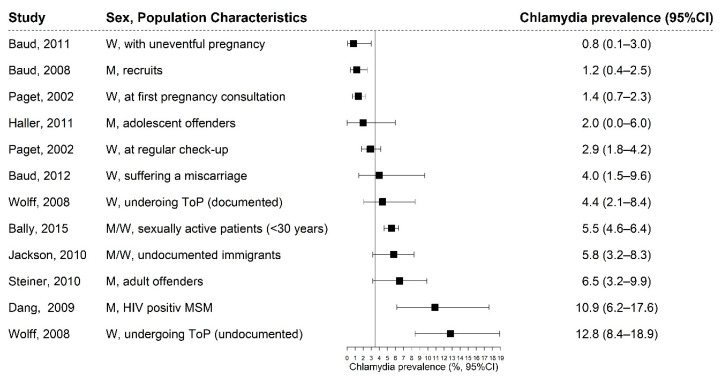
Forest plot of chlamydia prevalence from retrieved studies, with indication of the studied populations in Switzerland. The reference line at 3.6% represents the pooled chlamydia prevalence from nationally representative samples of women aged 18–26 years of four European countries assessed between 2004–2011 (3.6%, CI: 2.4–4.8). [29].

Chlamydia prevalence rates ranged from 0.8 to 12.8% (Figure 2). The highest rates were found among undocumented women undergoing ToP (12.8%, CI: 8.4–18.9), HIV-positive MSM (10.9%; CI: 6.2–17.6), male offenders (6.5%; CI: 3.2–9.9), undocumented immigrants (5.8%, CI: 3.2–8.3), and sexually active attendees of a cantonal sexual health network (5.5%, CI: 4.6–6.4) [17,25,26,27,28]. Those populations were at higher risk for chlamydia infections compared to nationally representative samples of women aged 18–26 living in four European countries with pooled chlamydia point estimates of 3.6% (CI: 2.4–4.8) (Figure 2) [29]. The lowest chlamydia rates were found among women having an uneventful pregnancy (0.8%; CI: 0.1–3.0), Swiss recruits (1.2%; CI: 0.4–2.5), and women attending a first pregnancy consultation (1.4%; CI: 0.7–2.3) [21,22,23]. Adolescent offenders were less likely to have a chlamydia infection compared to adult offenders (2 vs. 6.5%) [17,24] and undocumented women undergoing ToP were three times more likely to have a chlamydia infection than women with a legal residency permit (4.4 vs. 12.8%) [26]. Young women attending a cantonal sexual health network centre were 1.5 times more likely to have a chlamydia infection than their male peers (5.9 vs. 3.9%) [27].

### 3.3. Recommendations for Chlamydia Testing

In five included articles, the authors suggested specific chlamydia testing strategies among studied risk groups. Systematic chlamydia screening was suggested in women suffering a miscarriage [21], in HIV-positive MSM if unprotected anorectal intercourse was reported [28], and in undocumented women undergoing voluntary ToP [26]. Widespread surveillance among adult offenders [17] and no systematic screening among juvenile offenders was recommended [24].

## 4. Discussion

Nine studies assessed exclusive chlamydia prevalence in Switzerland and reported rates between 0.8 and 12.8%. Only one study was conducted at the population level, targeting Swiss men at the mandatory army recruitment day, whereas eight studies focused on specific risk groups. However, there is a lack of data on chlamydia prevalence rates among young, sexually active women living in Switzerland at the population level. Consequently, comparison of Swiss data with the pooled point estimates of nationally representative samples from four European countries of sexually active women aged 18–26 years of 3.6% (CI 2.4–4.8) is futile [29].

Six studies were conducted in the French-speaking part of Switzerland. This might indicate that regional differences exist regarding the awareness of chlamydia as a public health burden with francophone researchers and clinicians being more proactive. Whether some regions test more frequently for chlamydia compared to others is not inferable from published surveillance data. However, Schmutz et al. showed that, in a Swiss canton (Basel-Stadt), the number of performed chlamydia tests increased between 2002 and 2010 from 1395 to 3169. In parallel, the chlamydia positivity rate remained stable, indicating an increase in awareness [30].

According to our results, risk groups for chlamydia infections living in Switzerland include undocumented women undergoing ToP, HIV-positive MSM, offenders, undocumented immigrants, and young (<30 years) sexually active attendees of cantonal sexual health centres. Researchers explicitly recommend screening for three risk groups, including undocumented women undergoing ToP, women suffering a miscarriage, and anorectal testing among HIV-positive men reporting unprotected sexual intercourse. However, these risk groups are difficult to reach with a community pharmacy. First, women undergoing ToP or suffering a miscarriage are primarily cared for by their gynaecologists and offenders do not attend community pharmacies due to incarceration. Second, information such as sexual orientation is commonly unknown to pharmacists as well as HIV status, if no specific HIV treatment is listed in the medication history. Third, the legal status of customers, such as undocumented women, is commonly unknown to pharmacists. Although pharmacists have a pledge of secrecy and are therefore not allowed to report an illegal status to the police or migration office, undocumented women might be afraid to reveal their legal status to pharmacy staff upon asking. Further, undocumented individuals have the right to obtain health insurance in Switzerland. However, chlamydia tests are quite expensive (approx. EUR 90), and even undocumented individuals with health insurance must take a part of the costs by themselves. The fear of revealing their legal status and the healthcare costs make undocumented individuals hard to target through pharmacy-based testing service.

However, Bally et al. observed prevalence rates up to 5.9% among young (<30 years), sexually active women attending a sexual health network centre. A generalisation of these results suggests that young women seeking pharmacy-based sexual health services are likely to be affected by chlamydia. Today, Swiss pharmacists regularly counsel EC users on the risk of getting an STI while having unprotected sexual intercourse [31]. However, no data exist on how often Swiss EC users suffer from chlamydia infections. Thus, more studies are needed to determine prevalence rates in EC users and whether this group would profit from a pharmacy-based chlamydia testing service.

All included studies used nucleic acid amplification tests (NAATs) to detect chlamydia. NAAT is the gold standard according to CDC recommendations because of its superior sensitivity (80% to above 90%) and specificity (97–99%) compared to other test methods. [32,33] Most of the available NAAT tests are FDA cleared for the analysis of first-void urine samples and vaginal swabs. However, a test performed in a first-void urine sample from women detects up to 10% fewer chlamydia infections compared to vaginal or endocervical specimens. Additionally, the use of alternative testing methods such as direct fluorescent antibody tests, enzyme immunoassay tests, and serological tests is discouraged, because none of these tests is as sensitive or specific as NAATs. [32] Although the gold standard NAATs were used to detect chlamydia infections in our included studies, some infections might have remained undetected when, for example, women could choose between a first-void urine sample and a vaginal swab.

The only Swiss population-based study targeting recruits observed a very low chlamydia prevalence of 1.2% (CI: 0.4–2.5) compared to the pooled rate of 3.5% among four European countries [29]. The reasons for this difference might be that chlamydia infections in Swiss men peak at a higher age (25–34 years), while the targeted recruits had a mean age of 20.6 years. Moreover, this study was published in 2008, and extrapolation of the results is erroneous as chlamydia notification rates kept increasing over the past decade.

However, many industrialised countries including Australia, Canada, France, Malta, UK, and USA follow the CDC guidelines of opportunistically screening for chlamydia in sexually active women or both genders in the high-risk age group, or encouraging extensive testing such as Sweden. The worldwide prevalence of chlamydia at population level has been estimated at 2.9% (95% CI: 2.4–3.5) and specifically for Europe at 2.7% (95% CI: 1.9–3.6) [34]. In high-income countries such as Europe, chlamydia infections are most prevalent among young heterosexual adults with estimates of 3.6% (95% CI: 2.4–4.8) in women and 3.4% (95% CI: 1.9–5.2) in men aged 26 years or less at population level, and among MSM attending sexual health clinics [8,29]. In MSM, chlamydia positivity rates ranged from 2–5% for urethral and 6–9% for rectal infections [8]. A recently conducted cluster randomised controlled trial in rural Australia showed that a screening intervention could not significantly reduce the chlamydia prevalence in the intervention group (5.0 to 3.4%) compared to the control group (4.6 to 3.4%) [35]. Compared to the reported numbers, our prevalence results between 0.8–12.5% in Switzerland are not far away from the realities of other countries (prevalence reported between 2.9–9.0%). Reasons for similar findings are that, first, a large percentage of people infected with chlamydia are asymptomatic [10]. Second, some diagnostic methods have inadequate sensitivity below 80%; third, there is a large percentage of people who self-medicate, which delays testing [33].

Most of the identified risk groups in our review have also been the subject of studies in other countries. For example, juvenile offenders in the United States had higher chlamydia prevalence rates compared to Switzerland (USA: 5.9–14.4% vs. CH: 2.0%) [24,35]. Similarly, pregnant women in the Netherlands were at a higher risk of chlamydia infection (NL: 3.9% vs. CH: 0.8–1.4%] [21,30,36]. Conversely, MSM in California had a lower prevalence rate of anorectal chlamydia infections compared to their Swiss peers (USA: 7.9% vs. CH: 10.9%) [28,37]. However, in Switzerland, the study population was restricted to HIV-positive men, which might partly explain the discrepancy.

Multiple infections next to chlamydia are also observed in different populations. In a high-school population, co-infections with N. gonorrhoea (NG) occurred between 10–40% of the students [36]. In Switzerland, a co-infection rate of 32.5 and 23.6% for chlamydia and NG in women and men, respectively, has been reported by the University Hospital of Geneva [37]. Thus, individuals with chlamydia infections should also be screened for other STIs, including gonorrhoea, syphilis, HIV, and checked for the hepatitis B vaccination status [7].

Our study has several strengths. First, this is the first comprehensive collection of chlamydia point estimate studies in Switzerland, providing an overview of prevalence rates in Switzerland. Second, we focused on exclusive chlamydia screening studies and excluded those assessing a wide range of STIs. Although addressing all primary pathogens causing STIs would have been interesting because they include bacteria (Neisseria gonorrhoea, Treponema pallidum, chlamydia), parasites (Trichomonas vaginalis), and viruses (HIV, herpes virus—HSV, and Papilloma virus—HPV), this would have gone beyond the scope of identifying target groups for a pharmacy-based chlamydia testing service. By narrowing the focus, we aimed to collect data on patients most at risk for chlamydia infections and not for other STIs that might peak in a higher age class or in different risk groups. Third, interpreting Swiss studies within the context of international studies allowed us to detect shortcomings in the Swiss body of literature such as nationally representative population-based studies among women aged 15–24 years, and data regarding EC users. Fourth, we calculated a response rate for five of the nine included studies that ranged from 69 to 85%, which is remarkably high compared to the return rates of chlamydia self-test kits (12–51%) [4].

We acknowledge some limitations. First, we did not search for grey literature. However, we doubt that we missed extensive population-based studies or studies targeting pharmacy consumers that would have notably changed our conclusions. Second, six of nine studies were conducted from 2000 to 2010, when chlamydia notification rates were five times lower than today. Consequently, the results from these studies could not reflect the current situation of chlamydia infections in Switzerland. The absence of more recent studies underlines the fact that the body of literature is scarce in Switzerland and that more studies, including RCTs, are needed to specify the current guidelines concerning high-risk populations. This has been done by Catarino et al. who performed a retrospective analysis of chlamydia infections at the University Hospital of Geneva and suggested that chlamydia testing should be offered to young adults in high-risk settings such as the general emergency department and gynaecological consultations [37]. Third, all included studies were cross-sectional studies or cohort studies, with a poor level of evidence (evidence level 4) [38]. To add evidence to the body of literature, a pooled estimate of Swiss chlamydia prevalence would have been desirable. However, the studies were conducted in very heterogeneous populations, making a meta-analysis pointless. Thus, in the future, more robust study designs such as randomised controlled trials are needed to demonstrate the effectiveness of chlamydia testing among high-risk groups regarding health and economic outcomes in Switzerland with respect to the increasing threat of antimicrobial resistance.

## 5. Conclusions

Chlamydia infections are prevalent in Switzerland and in rates comparable to European countries. However, due to their unique situation, identified risk groups are difficult to reach via pharmacy-based testing services. The extrapolation of some findings suggests that customers seeking sexual-health-related services in community pharmacies might also be frequently affected by chlamydia. Thus, more studies are needed to identify suitable target groups for pharmacy-based chlamydia testing in Switzerland, including customers seeking sexual health-related services. In particular, women requesting EC already receive counselling about the risk of getting an STI during unprotected sexual intercourse. This counselling could be used as an opportunity to recommend chlamydia testing, if needed. Future approaches to identify target risk groups might include chlamydia prevalence testing among EC users attending community pharmacies. Further, to reinforce the pharmacist new role as gateway to health care [39], further pharmacy-based services might be developed to target individuals at particular risk for chlamydia infections in Switzerland such as individuals with multiple infections including HIV or hepatitis C.

## Figures and Tables

**Table 1:** Summary of the included studies, in alphabetical order by the first author.

Author, Year	Location	Population; Venue	Sex; Mean Age in Years (S.D.)	Specimen; Testing Method	Main Findings (Study Design, no. of Participants (% of Females if Applicable), Chlamydia Prevalence (%), Response Rate, Authors’ Conclusions)	Quality Assessment
Bally, 2015 [27]	Canton: Vaud, Valais	Sexually active patients < 30 years; centres of the cantonal public sexual health networks (*n* = 13) and infection disease outpatient clinics (*n* = 2)	W/M; 21.4 (N.R.)	First-void urine or self-applied low vagina swab for women according to their preferences; NAAT: PCR analysis with a commercial kit (Roche Diagnostics, Switzerland) or an in house validated test	Cross sectional study with 2455 participants (77% females); overall prevalence: 5.5% (95% CI: 4.6–6.4), W: 5.9% (CI: 4.8–7.0); M: 3.9% (CI: 2.3–5.5); response rate: 85%; opportunistic chlamydia testing is feasible and acceptable.	good
Baud, 2011 [21]	Lausanne	Women with an acute miscarriage (S.G.) or an uneventful pregnancy (C.G.); attending the emergency gynaecology ward or labour ward at the university hospital	W; S.G.: 33.3 (6.1) C.G: 31.5 (5.0)	Cervicovaginal swab, placenta, and serum; Placenta and cervicovaginal swab: NAAT: TaqMan real-time PCR analysis. Serum: tested for IgG and IgA using the Ridascreen IgG/IgA kit (R-biopharm, Darmstadt, Germany)	Cohort study with 386 participants; S.G.: 4.0% (CI: 1.5–9.6), C.G.: 0.8% (CI: 0.1–3.0); response rate: N.R.; women suffering a miscarriage should be screened for chlamydia to prevent a recurrence.	fair
Baud, 2008 [23]	Lausanne	Swiss recruits; the medical entrance examination at the Army recruitment centre	M; 20.6 (1.4)	First-void urine; NAAT: TaqMan real-time PCR analysis and genotyping of positive samples using ompA sequencing	Cross-sectional study with 517 participants; 1.2% (CI: 0.4–2.5); response rate: N.R.; chlamydia prevalence was extremely low in Swiss male recruits. Further research is required.	fair
Dang, 2009 [28]	nationwide	HIV-positive MSM enrolled in the SHCS having had unprotected anal intercourse in the past two years or symptoms of proctitis; outpatient clinics	M; median: 42 (N.R.)	Anal swab taken by study physician; NAAT: TaqMan real-time PCR. Genotyping of positive samples using ompA	Cross-sectional study with 147 participants; 10.9% (95% CI: 6.2–17.6); response rate: N.R.; routine anorectal chlamydia screening is suggested if unprotected intercourse is reported.	fair
Haller, 2011 [24]	Geneva	Juvenile offenders (>15 years); a juvenile detention facility	M; 16.2 (0.9)	First-void urine; NAAT: PCR analysis using Abott CT/NG reagent (Abott Molecular Diagnostics, Des Plaintes, IL, USA)	Cross-sectional study with 50 participants, 2% (CI: 0.0–6.0); response rate: 85%; results do not support systematic screening.	fair
Jackson, 2010 [25]	Geneva	Patients (18–50 years) with no legal residency permit or health insurance; a community Mobile Care Unit	M/W; 32.4 (8.0)	First-void urine; NAAT: PCR analysis using Abott CT/NG reagents (Abott Molecular Diagnostics, Des Plaintes, IL, USA)	Cross-sectional study with 313 participants (78.4% females); overall prevalence: 5.8% (CI: 3.2–8.3), M: 4% (CI: 1.3–10.6), W: 6.5% (CI: 3.7–11); response rate: 82%; access to testing should be ensured for this vulnerable population at risk.	fair
Paget, 2002 [22]	nationwide	Patients < 35 years having a first pregnancy consultation (S.G.) or a routine check-up (C.G.), private gynaecology practices	W; S.G. (median): 29 (N.R.) C.G. (median): 27 (N.R.)	Cervical swab; NAAT: LCR assay analysis (LCX, Abott Laboratories, Chicago, IL, USA)	Cross-sectional study with 1589 participants; S.G.: 1.4% (CI: 0.7–2.3), C.G.: 2.9% (CI: 1.8–4.2); response rate: N.R.; prevalence studies are important to assess chlamydia prevalence in addition to official laboratory reports, which underestimate the frequency of infections.	fair
Steiner, 2010 [17]	Geneva	Inmates (18–35 years); at a Swiss remand prison	M; 26.4 (6.4)	First-void urine; NAAT: PCR analysis using Abott CT/NG reagents (Abott Molecular Diagnostics, Des Plaintes, IL, USA)	Cross-sectional study with 214 participants; 6.5% (CI: 2.3–9.9); response rate: 80%; inmates are at particular risk for chlamydia infections and widespread surveillance is recommended.	good
Wolff, 2008 [26]	Geneva	Undocumented (S.G.) and documented (C.G.) women were undergoing voluntary ToP, the university hospital	W; S.G.: 28 (5.5) C.G.: 28.2 (7.5)	Cervical swab; NAAT: PCR analysis (test not further specified)	Cohort study with 378 participants; S.G.: 12.8% (CI: 8.4–18.9), C.G.: 4.4% (CI: 2.1–8.4); Response rate: S.G. (69%) and C.G.: (85%); Systematic screening among undocumented women undergoing ToP should be considered and access should be ensured.	fair

C.G.: control group; LCR: ligase chain reaction; MSM: men who have sex with men; NAAT: nucleic acid amplification tests; no.: number N.R.: not reported; PCR: polymerase chain reaction; S.D.: standard deviation; S.G.: study group; SHCS: Swiss HIV Cohort Study.

## Appendix A

**Table A1:** Search strategy.

Database	Search Terms
PubMed	chlamydia [MeSH Terms]
	chlamydia trachomatis [MeSH Terms]
	chlamydia infections [MeSH Terms]
	1 OR 2 OR 3
	screening [all Fields]
	mass screening [MeSH Terms]
	testing [all Fields]
	diagnosis [Subheading]
	diagnosis [all Fields]
	5 OR 6 OR 7 OR 8 OR 9
	Switzerland [MeSH Terms]
	Swiss [all Fields]
	11 OR 12
	4 AND 10 AND 13
	limit 14 to human
EMBASE	chlamydia [Emtree]
	chlamydia trachomatis [Emtree]
	chlamydia infection *
	1 OR 2 OR 3
	Screening [Emtree]
	testing
	diagnosis [Emtree]
	5 OR 6 OR 7
	Switzerland [Emtree]
	Swiss [Emtree]
	8 OR 9
	4 and 8 and 11
	limit 12 to human
Web of Science	1. chlamydia *
	2. testing *
	3. screening
	4. 2 OR 3
	5. Switzerland
	6. Swiss
	7. 5 OR 6
	8. 1 AND 4 AND 7
	9. limit 8 to human

* Terms entered with an asterisk at the end to identify various endings.

## Appendix B

**Table A2:** Quality assessment.

Criteria	Bally, 2015	Baud, 2011	Baud, 2008	Dang, 2009	Haller, 2011	Jackson, 2010	Paget, 2002	Steiner, 2010	Wolff, 2008
1. Was the research question or objective of this paper clearly stated?	yes	yes	yes	yes	yes	yes	yes	yes	yes
2. Was the study population clearly specified and defined?	yes	yes	yes	yes	yes	yes	yes	yes	yes
3. Was the participation rate of eligible persons at least 50%?	yes	NR	NR	NR	yes	yes	NR	yes	yes
4. Were all subjects selected or recruited from the same or similar populations (including the same time period)? Were inclusion and exclusion criteria for being in the study prespecified and applied uniformly to all participants?	yes	yes	yes	yes	yes	yes	yes	yes	no
5. Was a sample size justification, power description, or variance and effect estimates provided?	no	no	no	no	yes	no	no	yes	no
6. For the analyses in this paper, were the exposure(s) of interest measured prior to the outcome(s) being measured?	NA	NA	NA	NA	NA	NA	NA	NA	NA
7. Was the time frame sufficient so that one could reasonably expect to see an association between exposure and outcome if it existed?	NA	NA	NA	NA	NA	NA	NA	NA	NA
8. For exposures that can vary in amount or level, did the study examine different levels of the exposure as related to the outcome (e.g., categories of exposure, or exposure measured as a continuous variable)?	NA	NA	NA	NA	NA	NA	NA	NA	NA
9. Were the exposure measures (independent variables) clearly defined, valid, reliable, and implemented consistently across all study participants?	yes	NA	yes	yes	no	NR	NR	NR	NA
10. Was the exposure assessed more than once over time?	NA	NA	NA	NA	NA	NA	NA	NA	NA
11. Were the outcome measures (dependent variables) clearly defined, valid, reliable, and implemented consistently across all study participants?	yes	yes	yes	yes	yes	yes	yes	yes	yes
12. Were the outcome assessors blinded to the exposure status of participants?	NA	NA	NA	NA	NA	NA	NA	NA	NA
13. Was loss to follow-up after baseline 20% or less?	no	NA	NA	NA	NA	NA	NA	NA	NR
14. Were key potential confounding variables measured and adjusted statistically for their impact on the relationship between exposure(s) and outcome(s)?	yes	yes	yes	NA	no	yes	yes	yes	yes
Score (yes = 1, no = 0)	0.78	0.71	0.75	0.71	0.75	0.75	0.71	0.88	0.62
Quality rating (good, fair poor)	good	fair	good	fair	good	good	fair	good	fair
NR = not reported, NA = not applicable									
